# The diverse mechanisms and anticancer potential of naphthoquinones

**DOI:** 10.1186/s12935-019-0925-8

**Published:** 2019-08-02

**Authors:** Carolina Escardó Pereyra, Rafael Ferreira Dantas, Sabrina Baptista Ferreira, Luciano Pinho Gomes, Floriano Paes Silva-Jr

**Affiliations:** 1 Laboratório de Bioquímica Experimental e Computacional de Fármacos, Instituto Oswaldo Cruz, Fundação Oswaldo Cruz, Avenida Brasil 4365, Rio de Janeiro, Rio de Janeiro 21040-900 Brazil; 2 Laboratório de Síntese Orgânica e Prospecção Biológica, Instituto de Química, Universidade Federal do Rio de Janeiro, Ilha do Fundão, Rio de Janeiro, Rio de Janeiro 21949-900 Brazil

**Keywords:** Naphthoquinones, Natural compounds, Multi-target agents, Chemotherapy, Cancer drug discovery

## Abstract

Cancer is one of the leading causes of death around the world and although the different clinical approaches have helped to increase survival rates, incidence is still high and so its mortality. Chemotherapy is the only approach which is systemic, reaching cancer cells in all body tissues and the search for new potent and selective drugs is still an attractive field within cancer research. Naphthoquinones, natural and synthetic, have garnered much attention in the scientific community due to their pharmacological properties, among them anticancer action, and potential therapeutic significance. Many mechanisms of action have been reported which also depend on structural differences among them. Here, we describe some of the most relevant mechanisms of action reported so far for naphthoquinones and highlight novel targets which are being described in the literature. Furthermore, we gather some of the most impressive efforts done by researchers to harness the anticancer properties of these compounds through specifically designed structural modifications.

## Background

Non-communicable diseases (NCDs) are non-transmittable illnesses affecting all age groups and regions in the world. Cancer is considered one of the four types of NCDs—alongside cardiovascular diseases, chronic respiratory diseases and diabetes- and on its own, is one of the leading causes of death globally, with approximately 9.6 million cases estimated to occur in 2018 alone. Approximately 70% of all cases occur in low- and middle-income countries where cancer diagnostic and treatment services are insufficient [1].

Cancer is associated to uncontrollable cell growth and the different types known to date are classified according to the cell type that was initially affected. Surgery, radiation and chemotherapy are the three main approaches to cancer treatment. Damage of healthy tissues and cells are among the disadvantages of the first two approaches, as well as the fatigue from radiotherapy which may last for the duration of the treatment or months afterwards. Chemotherapy is the only approach which is systemic, meaning that the drugs circulate through the blood stream reaching cancer cells in all body tissues. It can annihilate both original tumor cells as well as metastasized ones. The drawback of this approach is that it has many side effects that affect the patient’s lifestyle and also, resistance may be developed by cancer cells [2].

**Fig. 1:**
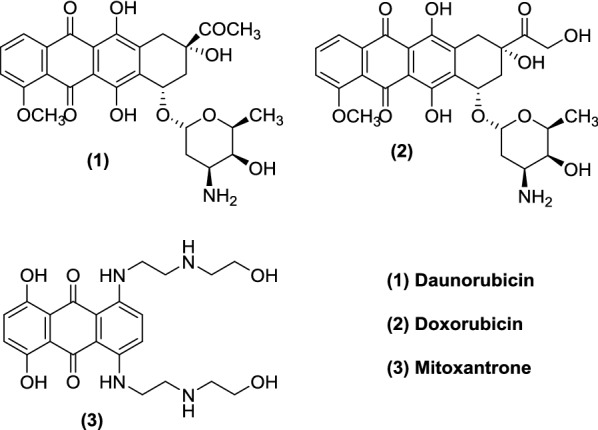
Chemical structures of anticancer drugs with the quinone moiety

Natural compounds and their derivatives are a good source of molecules to be tested for their anticancer properties. Among this group of compounds, quinones are ubiquitous in nature, occurring in animals, plants and microorganisms. They have a crucial role in the energy production of these organisms by providing essential links in the respiratory chain of the cells [3]. For a long time, they have been a source of cytotoxic compounds, being present in many drugs such as the anthracyclines daunorubicin (**1**), doxorubicin (**2**) and mitoxantrone (**3**), used clinically in the therapy of cancer (Fig. 1). Many other pharmacological activities have been reported, including their action as antiallergic [4, 5], antibacterial [6, 7], antifungal [6, 8], anti-inflammatory [4, 8], antithrombotic [9, 10], antiringworm [6], antiplatelet [4, 5] and antiviral agents [6, 10]. Cytotoxicity has been associated to the inhibition of the human DNA topoisomerase I and II [11–13] and production of reactive oxygen species (ROS) such as semiquinone and hydroxyl radicals by spontaneous or enzymatic reduction [14, 15].

In this review, we will focus on the pharmacology and the mechanisms of action reported up to date of one prominent type of quinones: naphthoquinones. Their anticancer activity has been widely reported in the scientific literature and many distinct mechanisms of action have been attributed to them. We will summarize some of the modifications which have been made to the core structure of the naphthoquinones in order to enhance their potential as anticancer agents and increase their selectivity for cancer cells or for one particular molecular target.

## Main text

### Cell cycle: checkpoints as therapeutic targets

Many small compounds, and among them natural products, can act at different stages of the cell cycle in order to stimulate cell replication, to arrest the cycle at one of its stages or to lead the cell into a state of depression, known as G0. In this state, the metabolic functions of the cell become inactive, namely transcription and protein synthesis. Proteins that act as controllers, allowing the cell to proceed or to halt its reproductive cycle have been properly identified and can therefore be used as therapeutic targets [16].

A molecule that acts on cell cycle proteins, whether by altering their levels or binding directly to them, can be a potential drug which could have a determining effect on cell proliferation. This becomes of high importance in diseases such as cancer, in which a failure in the control of this cycle or in the process of apoptosis (see below) leads to the installment of the sickness [17].

Four clearly distinguishable phases can be recognized in the cell cycle: G1, in which the cell prepares all its machinery towards DNA replication, S, which is the actual synthesis of new DNA identical to the one used as template, G2, when the cell now prepares for division and finally, M, known as mitosis, when the cell divides itself into two identical daughter cells. These phases are sequential and interdependent, i.e. they all need to take place in an orderly fashion to allow for correct cell duplication [16]. As mentioned above, there is also a stage known as G0 or latent state that is not a direct part of the sequence in the cycle. The entrance or the exit of the cell from this state is fundamental to the depression of its active functions  or to direct it to duplication.

The proteins responsible for the progression of the cell through the different stages are mainly kinases and phosphatases that act on each other producing their own activation or deactivation. Among kinases, the cyclin-dependent kinases (Cdks) play a fundamental role on the activation of other key proteins in the cycle through phosphorylation. These are modulated by another set of proteins known as cyclins [18]. This complexity allows for the search of many therapeutic targets and markedly increases the possibility of finding chemical compounds that permit the manipulation of the cycle at different stages. They can bind directly to a protein, leading to activation or blockage of its function as well downregulating or upregulating its expression.

Checkpoints in the cycle are mechanisms to ensure the cell that preceding phases required for successful cellular replication have been completed correctly. There are three key events that need to begin and finish flawlessly for the cell to divide, which are: DNA replication, DNA repair and chromosome segregation [19]. These are the stages at which many natural products can exert their effect and the damage created by them can lead to cell cycle arrest [16].

Accordingly, there are three main checkpoints at which the cell cycle can be stopped. In the G1/S transition, the cell needs to make sure that its machinery is ready for the actual replication of DNA, particularly that the template is not damaged. Eukaryotic cells cannot allow division unless DNA replication and damage repair are completed because these processes cannot occur after chromosome condensation has already happened and the cell is entering mitosis [19]. A key protein here is the mammalian p53 transcription factor and tumor suppressor. This factor senses the extent of the damage in the DNA and decides the fate of the cell, whether the damage is repairable and the cell can proceed to the next stage or the cell should undergo its death through apoptotic mechanisms [17]. In almost every tumor, p53 is inactivated due to direct mutations, mostly of missense type, in p53 gene (tumor protein p53, TP53) and/or to disturbances in its regulatory pathways (e.g. overexpression of negative regulators). Thus, the components of p53 system represent potential targets for cancer therapy [20].

Another checkpoint is during the other gap transition, G2, when the cell is preparing for division. Two enzymes play key roles in the correct replication of DNA: topoisomerases (topo) I and II. They are responsible for the regulation of the topology of DNA during replication, hence, their names. Topo I is always active whereas the topo II is mostly expressed during the G2 and M phases [16]. Pharmacological interference with any of these enzymes could lead to cell cycle arrest and the subsequent inhibition of cell division, representing another promising chemotherapeutic strategy.

Finally, there is a checkpoint at the mitosis stage, linked to the accurate spindle formation. This causes a delay of the anaphase in the mitosis through a signal generated by kinetochores when spindle fibers fail to attach during cell division [21].

### Apoptosis: signaling pathways and proteins involved

The term apoptosis for the process of programmed death carried out by the cell, was first coined by Kerr et al. [22]. It is a distinctive form of cell death, compared to necrosis, mainly because it is controlled and energy-dependent and no inflammation is associated to it [23]. Some authors even propose that the term necrosis should not be used to refer to cell death since it is linked to the degenerative processes that take place after the cell has already died. Additionally, apoptosis can occur on a single cell within an organism without affecting other neighboring cells. It is a process that normally takes place during the development of cells, to eliminate those that do not differentiate correctly, and also during aging [17].

The morphology of an apoptotic cell involves shrinkage, packing of organelles and pyknosis, a term used to refer to chromatin condensation and segregation [22]. Also, there is the formation of apoptotic bodies which contain cytoplasm from the dying cell and its packed organelles. These bodies may contain nuclear fragments in them or not, and they are subsequently phagocytized by macrophages, parenchymal cells or neoplastic cells. All these features prevent inflammation from taking place since the bodies are quickly removed and do not release their content into their surroundings [17].

The molecular processes that lead to apoptosis in a cell involve many proteins, being the family of the caspases the crucial ones. Caspases are proteases expressed in their proactive form in normal cells, cleaving substrates at aspartic acid residues. They can activate each other through their proteolytic activities giving rise to a biochemical cascade of events [17].

The two major classes of caspases are the initiators, caspase-2, -8, -9 and -10, and the executioners or effectors, caspase-3, -6 and -7 [24, 25]. In the latter group, caspase-3 is the most important one since it can be activated by any of the initiators, thus being an attractive target for anticancer therapy. There have been three other caspases identified, from which caspase-12 is of special interest for anticancer therapy. It mediates apoptotic pathways associated with endoplasmic reticulum stress that derives from oxidative stress [17], a phenomenon that could be caused by naphthoquinones when considered as anticancer agents (see next topic).

There are basically two signaling pathways in which apoptosis could occur: the extrinsic and the intrinsic one. In the former, there are transmembrane death receptors involved, such as the tumor necrosis factor (TNF) which brings the death signal from the exterior to the interior of the cell [26]. The intrinsic pathway depends on signaling inside of the cell and the events are mediated by the mitochondria. Signals could be of a positive or negative nature. The positive ones could be external or internal factors that could induce cell death, namely exposure to radiation, toxins, hypoxia, generation of free radicals, viral infections, etc. The negative signals are the absence of certain molecules that are in charge of suppressing death programs, leading to the initiation of the chain of events that ends in apoptosis [17]. There is also the perforin/granzyme pathway, which is a form of cell death mediated by T-cells [27]. All three apoptotic pathways aforementioned meet at the execution phase, where executioners caspases activate endonucleases and proteases leading to nuclear fragmentation and cytoskeletal degradation [17].

Focusing on the intrinsic pathway for anticancer therapy, the signals mentioned above cause changes in the inner mitochondrial membrane that lead to the loss of its transmembrane potential, loss of permeability and the release of pro-apoptotic substances that are normally retained into the cytosol [28]. These are cytochrome c, Smac/DIABLO and HtrA2/Omi [29, 30]. Cytochrome c activates procaspase-9, one of the initiator caspases, and the other two exert their pro-apoptotic action by inhibiting a group of proteins known as inhibitor of apoptosis proteins (IAP) [31, 32]. Another group of pro-apoptotic proteins that are released afterwards, once the cell is doomed to the path of irreversible death, are apoptosis-inducing factor (AIF), endonuclease C and caspase-activated DNase (CAD), mediating DNA fragmentation and condensation of chromatin. An excellent review of these events has been reported by Elmore, 2007 [17].

An important pro-apoptotic protein is p53. As mentioned above, its main functions include holding the cell cycle at the G1/S transition when it detects DNA damage, activating proteins that repair DNA and also leading the cell to its death if the damage is too severe [33]. This factor is mutated in 50% of human cancers, being the most mutated gene in humans with this disease [17].

The Bcl-2 (B-cell lymphoma 2 protein) family of proteins is also regulated by p53. These proteins are responsible for the regulation of the mitochondria-mediated apoptotic pathway and they can be either pro or anti-apoptotic. In a nutshell, they decide the fate of the cell, whether it goes on and dies or aborts the process. It is believed that Bcl-2 may exert its anti-apoptotic actions through antioxidant mechanisms [34].

### Endoplasmic reticulum stress (ERS): pro-survival and pro-death signaling pathways

The endoplasmic reticulum (ER) is a specialized organelle of eukaryotes that plays a major role in a myriad of physiological functions, including lipid synthesis, Ca^2+^ storage and detoxification. Moreover, ER is also responsible for the synthesis, folding and post-translational modifications of secreted and transmembrane proteins [35]. ER homeostasis can be disturbed by several factors, such as nutrient deprivation, excessive protein synthesis, imbalance in Ca^2+^ and ROS homeostasis, hypoxia and oxidative stress [35, 36]. If not resolved, these events may lead to ER stress (ERS), a condition characterized by the accumulation of misfolded proteins in ER lumen. ERS is associated to the physiopathology of many types of cancer [37] as well to metabolic [38], renal [39] and neurodegenerative disorders [40].

In order to overcome ERS, cells rely on a multipathway system called unfolded protein response (UPR) whose main goal is to restore ER proteostasis (i.e. protein homeostasis). UPR is regulated by three ER-resident transmembrane proteins (“stress sensors”): inositol-requiring enzyme 1 (IRE1α), activating transcription factor 6 (ATF6) and pancreatic ER kinase (PERK). The balance between pro-survival and pro-death mechanisms of UPR defines cells fate during ERS. Under mild ERS, UPR promotes cells survival, adaptation and resistance to death. In this scenario, ER homeostasis may be restored, and cell is kept alive. However, during chronic, severe and sustained ERS, UPR might be insufficient. In this situation, the pro-death mechanisms of UPR prevails over pro-survival ones which ultimately provokes cell apoptosis [41].

UPR participates in different aspects of cancer physiopathology and treatment [41]. In the microenvironment of tumors, ERS may be induced by extrinsic (e.g. hypoxia and nutrient deprivation) and intrinsic (e.g. oncogene activation and oxidative stress) factors [42]. In turn, cells activate UPR which ultimately leads to not only to cells survival, but contributes for the development of many cellular features that enable tumor growth and metastasis dissemination (the so-called hallmarks of cancer), including genomic instability, invasion, proliferation and angiogenesis [37, 43]. Such mechanisms have also been associated with resistance to chemotherapy and radiation. In this context, UPR modulation represent a useful approach to develop new anticancer drugs. In fact, several natural and synthetic compounds induce apoptosis of cancer cells by either blocking UPR pathway (e.g. inhibiting PERK activity, decreasing the expression of ERS-related proteins) or inducing chronic ERS [41, 42].

### Naphthoquinones: occurrence in nature and structural characteristics

**Fig. 2:**
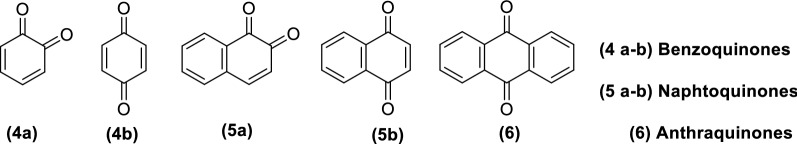
Basic structural cores within the quinone family

Quinones could be chemically classified into three different classes regarding the type of aromatic system that supports the quinone ring (Fig. 2): benzoquinones (a benzene ring) (**4a** and **4b**), naphthoquinones (a naphthalene ring) (**5a** and **5b**) and anthraquinones (an anthracene ring, linear or angular) (**6**). Depending on the position of the carbonyls within the ring system, one could have different quinones. As an example, naphthoquinones could have two different arrangements for their carbonyl groups: 1,2-naphthoquinones (**5a**) with neighboring functional groups or 1,4-naphthoquinones (**5b**) with a space of two carbons between carbonyls. These isomers have notably different pharmacological actions due to the difference in their physicochemical properties [13].

Quinones are highly reactive compounds with an application as natural or synthetic dyes. Widely distributed in nature, they can be found in several families of higher plants (Table 1). Naphthoquinones are particularly ubiquitous in the families *Bignoniaceae*, *Verbenaceae* and *Proteaceae*, though their major occurrence is on the former, specifically on the genus *Tabebuia* [13]. They are also present as secondary metabolites of microorganisms.

**Table 1:** Families of higher plants from which naphthoquinones have been isolated. Adapted from López et al. [44]

Families of higher plants	
*Ancistrocladaceae*	*Gentianaceae*
*Avicenniaceae*	*Iridaceae*
*Balsaminceae*	*Juglandaceae*
*Bignoniaceae* ^a^	*Lythraceae*
*Boraginaceae*	*Nepenthaceae*
*Dioncophyllaceae*	*Plumbaginaceae*
*Droseraceae*	*Proteaceae* ^a^
*Ebenaceae*	*Scrophulariaceae*
*Euphorbiaceae*	*Verbenaceae* ^a^

^a^Most common families

**Fig. 3:**
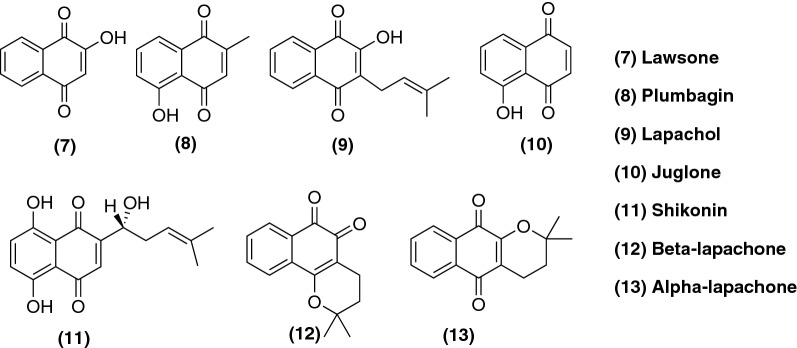
Examples of naturally-occurring naphthoquinones

Lawsone (**7**), plumbagin (**8**), lapachol (**9**), juglone (**10**) and shikonin (**11**) are naturally-occurring naphthoquinones, isolated from plants, which show many biological and pharmacological properties as well as being used as models for further structural modifications, as we will see later on this review [44]. Isolated from the root tissues of *Lithospermum erythrorhizon* and known in Chinese traditional medicine for many years for the treatment of sore throat, burns, cuts and skin diseases, shikonin (**11**) acts in a variety of molecular targets that are associated to the genesis of cancer, such as the upregulation of p53 and increased expression of apoptosis regulator BAX (Bax) [45, 46]. Besides, shikonin (**11**) shows similar potency towards cells which are drug-resistant and those which are drug-sensitive, as further discussed in the next section. Furthermore, β-lapachone (**12**) also occurs naturally and is isolated from the bark of lapacho tree (*Tabebuia avellanedae*) [47] as well your positional isomer, α-lapachone (**13**) (Fig. 3). A good review on naphthoquinones obtained from natural sources and their action was published by Nematollahi et al. [48].

### Mechanisms of action of naphthoquinones as anticancer agents

Naphthoquinones are considered privileged structures in medicinal chemistry, a term coined by Evans et al. [49] to highlight substructures associated to diverse pharmacological activities while allowing easy chemical modifications. This makes them suitable scaffolds for the synthesis of new compounds that reproduce the same known actions or lead to new ones, also improving their absorption, distribution, metabolism, excretion and toxicity (ADMET) properties and even their pharmacodynamics.

The focus of this review is on the anticancer properties of naphthoquinones. Many mechanisms have been reported in the literature about how this class of compounds exert their action on cancer cells and others have been proposed but not yet completely elucidated. Hence, it is likely that they kill or induce cell death by more than one mechanism, which will be described in the present review.

#### Generation of ROS and oxidative stress (DNA damage)

In eukaryotic cells, ROS are generated as byproducts of metabolism in mitochondria, as well in other cellular organs that consume high levels of oxygen. Some of the most reactive ROS have unpaired electrons (free radicals), such as O_2_^−^ and ·OH. Although H_2_O_2_ is not a free radical, it has the ability to diffuse through cell membranes, thus affecting sites other than the one where it was produced. Moreover, it can be converted to ·OH by the so-called Fenton reaction. Low concentrations of ROS are necessary for certain functions in the cell to take place normally, for example the folding of new proteins in the ER and the control of caspase activity during apoptosis [2, 50].

Under specific conditions ROS may have deleterious effects on cells. The damage depends on their intracellular concentration and on the equilibrium with endogenous antioxidant species. If this equilibrium is lost, oxidative stress takes place in the cell causing damage to molecules such as proteins, lipids, DNA and RNA [50]. Regarding DNA damage, which is the one mostly associated with carcinogenesis, three types have been reported: base modification, strand breakage and DNA–protein cross-linkage. The base 2-deoxyguanosine is particularly affected by the hydroxyl radical and is one of the major damages that occurs in vivo causing mutations to DNA [2]. The derivative 8-hydroxy-2′-deoxyguanosine is considered a biomarker of oxidative stress and carcinogenesis [51].

**Fig. 4:**
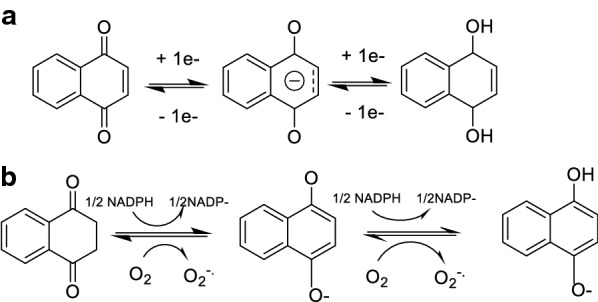
Redox properties of naphthoquinones. **a** One-electron reduction of the naphthoquinone core, first to the radical semiquinone and then to the hydroquinone. **b** Reduction of the naphthoquinone core in a biochemical context in the presence of NADPH under aerobic conditions, with the resulting production of the superoxide radical anion. It is important to consider that semiquinones cannot be reoxidized to quinones without oxygen and, therefore, can accumulate in the cell in anaerobic conditions [2]

Naphthoquinones bear the ability to accept one or two electrons to form semiquinones and hydroxyquinones, both highly reactive species (Fig. 4). These species can then be oxidized again by molecular oxygen generating ROS. Its direct implication in apoptosis has been reported by Stangel et al. [52]. Also, naphthoquinones can participate in biochemical reactions being reduced to the semiquinone, a free radical, and subsequently to the hydroxyquinone (Fig. 4). Under aerobic conditions, one-electron reductions predominate, resulting in free radical intermediates [53]. They are carried out mainly by the detoxifying enzyme cytochrome P450 reductase and other flavoprotein enzymes [44]. As an alternative pathway for the activation, two-electron reductions can take place followed by its inactivation through subsequent glucuronidation and/or sulfation or by conversion into an alkylating intermediate. This pattern is believed to be the preferred one in anaerobic conditions [53] and the enzyme responsible for them is NQO1 (NADPH: quinone oxidoreductase 1), which is abundantly expressed in cancer tissues [54].

Cancer cells are believed to have greater concentration of ROS compared to normal cells. They also have higher metabolic demands and activity, thus requiring more ATP. This leads to a major formation of superoxide radical anions through the electron transport chain. It is also believed that this higher, but non-lethal concentration of ROS, promotes tumor growth through cancer cell survival even when damaging DNA, since some of these damages could be advantageous for the cancer cell [55]. This imbalance could be used as a target for chemotherapy since the production of even higher amounts of radical species in the cell can make the situation even more critical leading the cell to its death [2]. Here is where naphthoquinones can have one of their actions as anticancer agents and this would also serve as an explanation to their selectivity towards cancer cells rather than normal ones, since their oxidative stress should not be naturally imbalanced. For some naphthoquinones, such as β-lapachone (**12**), selectivity may also be due to overexpression of NQO1 in cancer cells since these compounds require this enzyme to become fully reactive [56].

A class of enzymes which have been reported to be targeted by ROS are cysteine proteases. The thiol of the catalytic Cys residue in them would undergo oxidation by ROS to the sulfenic acid leading to the inhibition of their activity [57, 58]. Within this type of enzymes, ubiquitin-specific proteases 1 and 2 (USP1 and USP2) are linked with DNA damage repair. Particularly, USP2 has been associated with apoptosis resistance by cancer cells through stabilization of Fas and cyclin D1 [59]. It has been reported that both enzymes are susceptible to ROS and are inhibited by the natural *ortho*-quinone β-lapachone (**12**), suggesting that they are interesting targets for the development of compounds for cancer therapy. Also, a correlation was established between redox potentials of several *ortho*-quinones and their inhibition effect on the enzyme. Interestingly, when they tested the best candidates of the series on DU145 prostate cancer cells, which overexpresses USP2, the compounds did not exhibit potent activity [58]. The same group which reported these results, later on published a second article focusing on *para*-quinones suggesting that they also inhibit USP2 through redox cycling [59]. This finding was unexpected since *para*-quinones are not regarded as redox cyclers and are believed to exert their action precisely through different mechanisms of actions that do not depend so much on ROS formation [60].

**Fig. 5:**
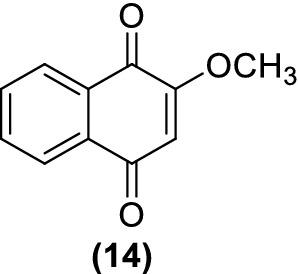
Structure of 2-methoxy-1,4-naphthoquinone (MNQ)

A recent work [61] investigated the molecular mechanism and intracellular targets of plumbagin (**8**) related to its cytotoxic effect in HepG2 hepatocellular carcinoma cells. Target identification was carried out by similarity ensemble approach (SEA), interestingly obtaining the result that most of candidate target proteins are involved in redox signaling. Among these proteins, some of the most significant were mitogen-activated protein kinases (MAPK), thioredoxin reductase and glutathione reductase. On the last two, plumbagin (**8**) acted by preventing reaction with their intracellular targets and by direct inhibition of the protein, respectively. Ong et al. [62], studied the cytotoxicity of 2-methoxy-1,4-naphthoquinone (MNQ) (**14**) (Fig. 5) a naturally-occurring naphthoquinone extracted from *Impatiens balsamina*, in A549 lung adenocarcinoma cells, reporting for the first time its action on the JNK (c-jun-NH2-kinase) and MAPK signaling pathways, triggered through the generation of ROS.

##### i. NQO1: its role in cancer and as quinone detoxifier

NQO1 is a two-electron reductase mainly located in the cytosol, which can use either nicotinamide adenine dinucleotide reduced (NADH) or nicotinamide adenine dinucleotide phosphate reduced (NADPH) as electron donors. This enzyme has an important role in the protection of the cell against natural and exogenous quinones. It is a homodimer made up of monomers of 247 residues with two flavine adenine dinucleotide (FAD) cofactors at each active site of each monomer [63], and it is reported to be inhibited by dicoumarol (**15**) [64]. When reducing quinones to hydroquinones in a single step, it yields substrates for Phase II conjugation and subsequent elimination and it also bypasses the toxic radical intermediates that occur in the one-electron reduction [65]. Both, *ortho*- and *para*-quinones are believed to be substrates of this enzyme [66]. For instance, β-lapachone (**12**) is one of its substrates, being eliminated metabolically by NQO1 through quinone reduction and subsequent glucuronidation [67]. The hydroquinones produced by NQO1 reduction are not always stable, being able to react with molecular oxygen to form semiquinones, producing ROS which can generate oxidative stress, or forming the semiquinones through comproportionation reactions [68]. The hydroquinone formed through this mechanism can act as an alkylating agent for nucleophilic sites, including DNA, which can lead to irreparable damage. NQO1-directed anticancer agents are designed to target this issue. It is important to highlight that the stability of the final hydroquinone produced will determine the role of NQO1 as a detoxifier or bioactivating enzyme [65]. Also, on this note, it has been reported that some quinones can redirect NQO1 to a ‘futile redox cycle’ in which the unstable final hydroquinone re-oxidizes in a spontaneous manner into parental compounds. This results in very high levels of superoxide anion which can then be metabolized by the enzyme superoxide dismutase into hydrogen peroxide, causing oxidative stress and damage [69].

**Fig. 6:**
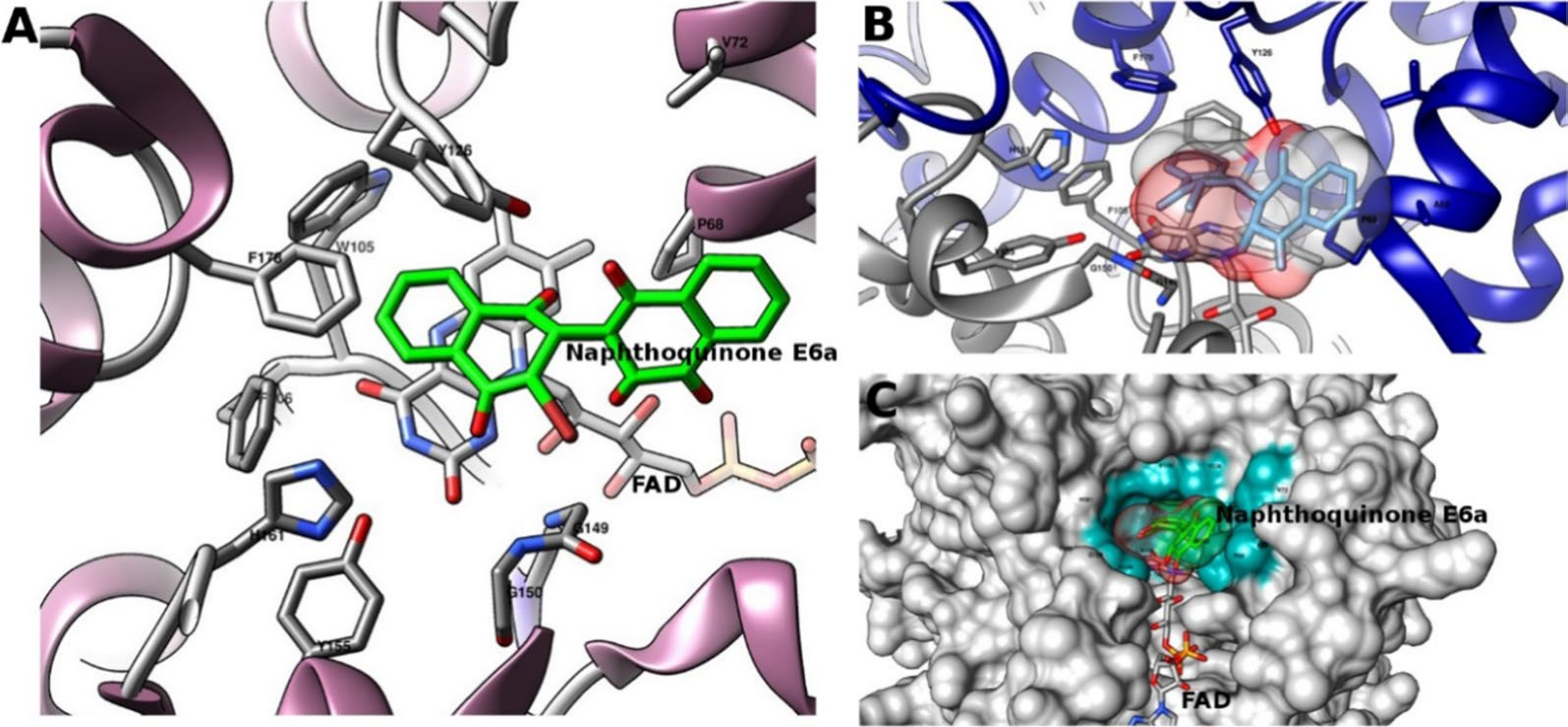
Visualization of naphthoquinone E6a (**16**) and its interaction with NAD(P)H dehydrogenase (pdb code: 5EAI). **a** The naphthoquinone is represented in green, FAD and the protein in cartoon and side chains of residues with a distance less than 5Å are highlighted. **b** Protein chains highlighted in blue and gray and the surface of the naphthoquinone in colors per heteroatom. **c** Representation of the protein’s surface and the binding site of the naphthoquinone together with FAD. The cyan surface represents the residues which belong to the binding site with the distance criterion to the naphthoquinone (surfaces were calculated according to Sanner et al. [73]). Molecular modeling graphs were produced with the package UCSF Chimera [74]

NQO1 is expressed in many tissues, particularly in those which require a high level of antioxidant protection. Nevertheless, it has been widely reported that NQO1 is overexpressed in cancer tissues, which would serve as a justification for the selectivity of agents targeting this enzyme [70, 71]. NQO1 could serve as a target for cancer therapy through two different mechanisms: its inhibition can lead to cell growth suppression since the normal function of this enzyme in the cell is to protect them from mutagenic, carcinogenic and cytotoxic effects derived from quinones. On the other hand, NQO1 can also be used as a tool for activating quinone-like compounds through reduction. A recent study carried out by Pidugu et al. [63], reported for the first time the direct interaction of a dimeric naphthoquinone, E6a (**16**), with NQO1 through the determination of the crystal structure of the complex with this compound bound to the enzyme, also providing evidence that this interaction directly affects the redox state of the FAD cofactors (Fig. 6) [63].

Photodynamic therapy is one of the approaches for cancer treatment which has been clinically approved and is gaining relevance in the last years. It involves the administration of a compound which acts as photosensitizer (e.g. 5-aminolevulinic acid, and its methylated derivative) followed by illumination with light of a specific wavelength, locally. Selectivity derives from the ability of the sensitizer to accumulate in the tumor and the precise delivery of light specifically where it is needed and one of its main advantages is that it is minimally invasive. It has been reported that expression of NQO1 in human breast cancer MCF-7c3 cells is induced after photodynamic therapy and that, consequently, the sensitivity of cancer cells to treatment with β-lapachone (**12**) increases [72]. These findings suggest that the combination of this type of therapy with β-lapachone (**12**), or any other substrate of NQO1, would be synergistic and a good field of study for the development of new therapies.

##### ii. Food for thought

It has been established that one of the main mechanisms of actions for naphthoquinones is the generation of ROS. What could be, then, the explanation for not finding direct correlations between redox potentials calculated in vitro and the biological effect? In our particular experience, those redox potentials were positive, which implies a spontaneous reduction of the compounds, meaning that they would be producing ROS even in the absence of reductases, but probably this amount of ROS is not enough for killing the cell. However, it is important to consider that redox potentials calculated from cyclic voltammetry cannot be considered as exact indications of standard potentials and cannot be directly linked to the potential which would be observed for the same molecules under physiological conditions [75]. One must bear in mind that the environment in which the redox reaction takes place will influence its outcome dramatically and when it comes to the cell, characteristics such as lipophilicity, oxygen partial pressure, etc. might vary a lot compared to aqueous solutions [53]. Nevertheless, there are some groups of scientists which have been able to prove correlations between reduction/oxidation (redox) potentials of some naphthoquinones and the biological effect observed [76, 77], but in our experience this is not always the case.

Maybe ROS generation by naphthoquinones depends on the presence of the enzymes which reduce them to generate the actual amount of ROS which is lethal for the cell. Would this be the case, overexpression of these enzymes would be crucial for naphthoquinones to exert their action, which would also serve as an explanation towards their selectivity, since enzymes such as NQO1 are reportedly overexpressed in cancer tumors. On the same note, it is also probable that not all naphthoquinones work mainly through this mechanism, but they might prefer others depending specifically on the characteristics of their chemical structure. This backs up the idea that not all naphthoquinones are substrates for the same enzymes.

#### ERS and induction of apoptosis

There is an interplay between the level of Ca^2+^ in the cell, ERS mediated by ROS generation and mitochondrial function that can lead to apoptosis. Gara et al. [45], have recently proven the role of shikonin (**11**) in inducing cell death through ERS in prostate cancer cell lines (DU-145, PC-3), having a direct effect on the production of ROS and an increase in the amount of Ca^2+^ in the cell.

It has also been reported that shikonin (**11**) directly inhibits tumor proteasome in studies performed in vitro (murine hepatoma H22 and leukemia P388; human prostate cancer PC-3 cells) and in vivo (murine H22 allografts and PC-3 xenografts) [78]. Proteasomes are protein complexes present in all eukaryotic cells whose main function is to degrade damaged or unfolded proteins by proteolysis. The action of shikonin (**11**) on the tumor proteasome could lead then to ERS generated by an excess of unfolded/misfolded proteins in the cell because of lack of their clearance [45].

An important recent study [79] has shown that while shikonin (**11**) triggers the ERS response in human glioblastoma stem cells as a way of exerting its cytotoxic effect, this can also generate the activation of pro-survival pathways that may compromise its anticancer activity. They reported that while ERS is involved in the antitumor activity of shikonin (**11**) it also compromises its cytotoxicity via upregulation of the JNK/c-Jun pathway and that inhibition of ERS in cancer cells could potentiate cytotoxic effects.

Recent studies demonstrated the role of NQO1 in the cytotoxicity of a dual therapy with β-lapachone (**12**) and ionizing radiation in NQO1^+^-MDA-MB-231 human breast cancer cells, and the induction of mitochondria-mediated cell death through ERS induced JNK pathway activation [80]. On this note, uridine diphosphate-glucuronosyltransferases (UGTs), are important phase II metabolic enzymes which help in the elimination of the products of the two-electron reduction of NQO1 substrates, acting as detoxifiers. In their absence, the compound could return to its original form by oxidation, producing ROS in the way. Studies with human colon cancer cells (HT29 and HCT116) exposed to β-lapachone (**12**) have proven that UGTs glucuronidate the toxic catechol that is the product of its reduction by NQO1. Hence, the action of UGTs could be the reason why many drugs targeting NQO1 have shown chemoresistance [67].

**Fig. 7:**
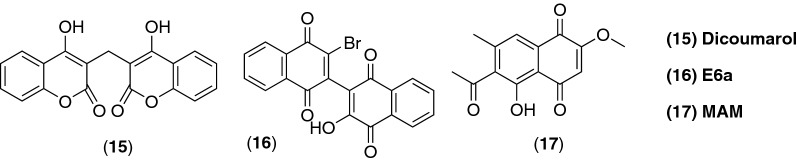
Chemical structures of dicoumarol, dimeric naphtoquinone E6a and MAM (2-methoxy-6-acetyl-7-methyljuglone)

Another very recent study has reported that 2-methoxy-6-acetyl-7-methyljuglone (MAM) (**17**) (Fig. 7), a natural derivative of juglone (**10**), isolated from *P. cuspidatum*, induced apoptosis in a caspase-dependent manner on some human cancer cell lines like MCF7 (breast) and B16-F10 (melanoma) and necrosis in A549 (lung) cells. These effects were mediated by the production of hydrogen peroxide inducing the JNK/iNOS/NO (c-jun-NH2-kinase/inducible nitric oxide synthase/nitric oxide) pathway [81].

#### Regulation of the tumor suppressor factors p53 and p73

Activation of p53 factor seems to be a potential target in anticancer therapy and an important aspect in the pharmacology of naphthoquinones, since the modulation of the factor itself or the molecules that it targets, can help in the modulation of the apoptotic intrinsic pathway. There is another tumor suppressor factor, p73, that can act on the targets of p53, particularly on PUMA, p21 and p16INKA4 [82]. This factor is usually kept at low levels in basal conditions and its concentration is augmented when the cell is stressed [83]. In addition to this, inverted CCAAT box binding protein of 90 kDa (ICBP90) is a nuclear protein which promotes the activity of topo IIα by binding to part of a known sequence of its gene promoter [84]. This protein is regulated by both tumor suppressor factors already mentioned, p53 and p73, and it is believed to be overexpressed in various types of cancer [82].

A recent study proved that shikonin (**11**) produces the up-regulation of the p73 factor in human cervical (HeLa) and breast (MCF-7) cancer cells, thus down-regulating the anti-apoptotic ICBP90 and re-expressing p16INK4A, one of the targets of p53 and also a pro-apoptotic factor [82]. Also, it has been reported that shikonin (**11**) can activate p53 in response to DNA damage, reducing the expression of cdk4, leading to apoptosis in human malignant melanoma A375-S2 cells and also through the upregulation of Bax and downregulation of Bcl-2 [85].

Plumbagin (**8**), isolated from the roots of *Plumbago scandens* L., has been reported to upregulate the expression of p53 in a panel of human brain cancer cell lines, inducing cell cycle arrest at the G2/M stage, and altering the Bax/Bcl-2 ratio [86]. Also, it increases the expression of apoptosis markers caspase-3 and -7 in human HepG2 hepatocellular carcinoma cells [61].

#### Inhibition of the DNA topoisomerases

As mentioned before, naphthoquinones have been identified to inhibit both types of topoisomerases present in the eukaryotic cells: I and II, being the latter mostly associated with their cytotoxic activity and the target for many anticancer agents. Both enzymes break DNA at the phosphodiester bond using a catalytic tyrosine residue and are critical for the correct functioning of the cell. Any alteration in their balance is enough to induce apoptosis [13].

The catalytic function of topo II is essential for the maintenance of the topology of the DNA molecule during replication, transcription and recombination. The enzyme has the ability of introducing a transient double-stranded break on the DNA molecule by binding non-covalently to it and is attached in a covalent manner to the 5′-phosphate of the DNA molecule. After ATP is bound, a second strand of DNA passes through the break and the enzyme reseals it. These events are known as pre-strand and post-strand passage cleavage, respectively [87].

**Fig. 8:**
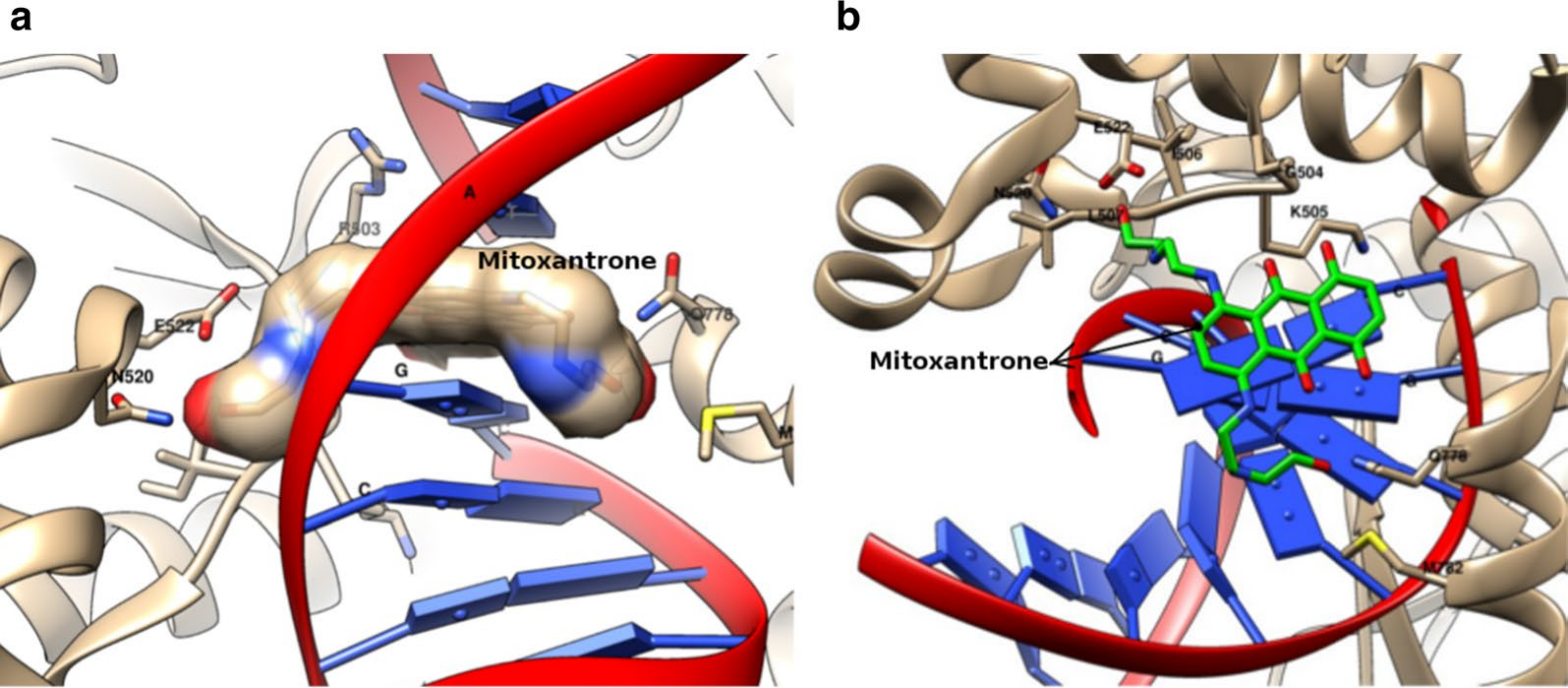
Representations of the complex human β topoisomerase II, mitoxantrone and DNA (pdb code: 4G0V). **a** Representation of the view on the side of mitoxantrone which is colored by atomic type. Side chains of the residues highlighted and named with the criterion of distances less than 5Å; DNA in cartoon. **b** Representation of the top view of mitoxantrone (carbons in green) complexed with DNA and the side chains of the residues (surfaces were calculated according to Sanner et al. [73]). Molecular modeling graphs were produced with the package UCSF Chimera [74]

There are two classes of inhibitors according to their mechanism. ‘Poisons’ are those which stabilize the covalent intermediates of the enzyme, usually a ternary complex involving DNA, the enzyme and the compound [88]. Those which work at any other stage of the catalytic cycle are simply called “catalytic inhibitors”. Doxorubicin (**2**), a well-known anthracycline used clinically for the treatment of cancer, is classified as a “poison” [89] and so are the majority of the compounds which inhibit these enzymes. Another example of a “poison” is mitoxantrone (**3**) which is depicted in Fig. 8 bound to topo II-β in the presence of DNA.

Eleutherin (**18**), a naturally-occurring compound isolated from the bulb of *Eleutherine americana*, has been identified as a reversible catalytic inhibitor of topo II by Krishnan and Barstow [87]. They also proved the in vitro inhibition of topo II by α and β-lapachone (**13**, **12**) and classified them as irreversible catalytic inhibitors. The mechanisms proposed are described in detail in both excellent publications on the topic by the same group in the years 2000 and 2001 [87, 90].

Type I topoisomerase has received less attention regarding the action of naphthoquinones and their inhibition of the enzyme. The enzyme received its name because it introduces single-strand breaks into the DNA molecule [88]. It has been reported that shikonin (**11**) is an inhibitor of the enzyme at low concentrations (IC_50_ = 2 µM) in studies carried out by Zhang and collaborators, 2013 [91]. β-lapachone (**12**) was first reported as a topo I inhibitor but studies on yeasts lacking expression of the enzyme showed that the compound could still suppress their growth [87]. Some naphthoquinone derivatives have been synthesized and their inhibition of the enzyme has been proven [11, 12], but most of the work that has been dedicated to it is in relation to camptothecin derivatives, a quinolone alkaloid, which finally resulted in two well-known anticancer drugs: topotecan (**19**) and irinotecan (**20**).

Apart from the inhibitors described so far, there is another class of compounds which act as dual inhibitors. They could be classified in three types: (i) drugs which bind directly to DNA, usually by intercalation; (ii) hybrid molecules rationally designed by linking inhibitors of both enzymes, type I and II, or by linking pure inhibitors to DNA-interactive carriers, and (iii) compounds which recognize structural motifs present in both enzymes [92]. Saintopin (**21**), a fungal secondary metabolite, was the first compound reported as a dual inhibitor of the type (i) described above, binding weakly through intercalation to substrates of both types of enzymes and inhibiting their catalytic activity [93].

Some of the problems related to the classic inhibitors are resistance developed by the cells, which causes clinical failure in long-term therapies [94], and the fact that some topo II inhibitors have caused secondary malignancies since they can trigger chromosomal translocations which may lead to a specific type of leukemia [95].

**Fig. 9:**
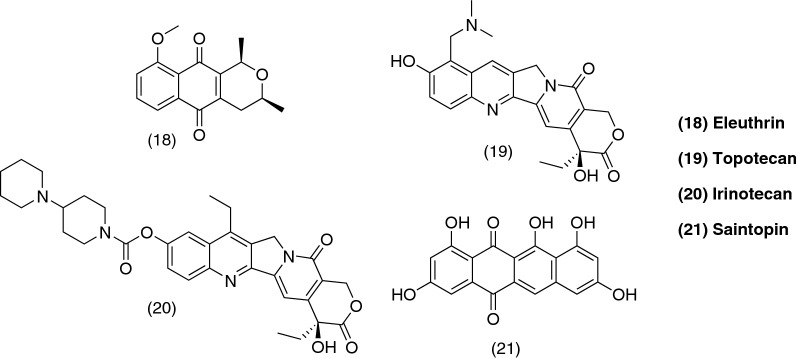
Chemical structures of quinone-like topoisomerase inhibitors

A summary of the types of inhibitors and the most notable molecules which have been developed/discovered is on Table 2 and Fig. 9.

**Table 2:** Table of quinone-like topoisomerase inhibitors

Topoisomerase II	
Poisons	Doxorubicin, MIX
Reversible catalytic inhibitor	Eleutherin
Irreversible catalytic inhibitors	α and β-lapachone
Topoisomerase I	
Shikonin, camptothecin derivatives	
Dual	
Saintopin	

*MIX* mitoxantrone

#### Other targets and mechanisms of action reported: recent advances in the anticancer pharmacology of naphthoquinones

Recently, novel targets for naphthoquinones as anticancer agents have been reported in the pursue of a better understanding of their action on cancer cells and the apoptotic pathways they may trigger. Inflammation has now been widely recognized as an important factor in tumor initiation and progression, leading to oncogenic transformation. Genetic and epigenetic changes in cancerous cells also generate the inflammatory microenvironment the tumor needs in order to survive and progress [96]. It is suggested that the signal transducer and activator of transcription (STAT) family proteins, particularly STAT3, selectively induce and maintain the inflammatory microenvironment, at the initiation as well as during progression of the tumor [97]. It is linked to inflammation-associated tumorigenesis initiated by genetic alterations in cancerous cells as well as by many other external factors such as chemicals, UV radiation, stress, cigarette smoking and infection [96]. This type of signaler undergoes phosphorylation at a specific tyrosine residue (Tyr^705^ for STAT3) followed by homo/hetero-dimerization, translocation to the nucleus and activation of the expression of specific genes. STAT3 works as a homodimer or a STAT1-STAT3 heterodimer [98]. Three domains within the protein have been identified as suitable for targeting compounds: amino-terminal, DNA binding and SH2, which is involved in receptor-binding and dimerization [99]. Kortylewski et al. reported that STAT3 could be used as a target for cancer therapy and that its removal, even under chronic inflammatory conditions, inhibits carcinogenesis and the growth of established tumors. Also, mice reconstituted with S*tat3*-deficient immune cells can generate a potent anti-tumor immune response. This group were also the first to report that STAT3 is persistently activated in immune cells associated to a tumor, which leads to the suppression of innate and adaptive immune responses [100].

Bhasin and collaborators, 2013 [99], worked on the synthesis of anthraquinone and naphthoquinone derivatives using as a model STA-21 (**22**), which was the first reported inhibitor of the SH2 domain of STAT3 [101], and maintaining its quinone moiety. They tested their antiproliferative activity in human cancer cells lines of prostate (DU-145) and colon (HT-29) obtaining positive results, which suggests that STAT3 could be one of the many targets of this class of compounds and a new line to exploit.

Joo et al. [102], focused on the action of plumbagin (**8**) on the STAT3 signaling pathway in a gastric cancer cell line (MNK-28) and suggested that it negatively modulates its activity via dephosphorylation rather than protein degradation. They also suggested that plumbagin (**8**) inhibits cell proliferation in a time- and dose-dependent manner, as well as migration and invasion and induces apoptosis in MKN-28 cells.

Mutation and activation of the epidermal growth factor receptor (EGFR) has been detected in many solid tumors. Also, nuclear factor kappa B (NF-κB) has a crucial role in the induction of the expression of inflammatory mediators as well as being the main transcription factor in many immune responses [103]. Unsurprisingly, signaling by STAT3 and NF-κB are highly interrelated [96]. Tian et al. [104], demonstrated the role of shikonin (**11**) on the inhibition of the EGFR-NF-κB signaling pathway on epidermoid carcinoma cells, A431. It decreased the phosphorylation of EGFR and STAT3 in a concentration-dependent manner (2.5 µM, 5 µM and 10 µM were tested). Moreover, Zhao et al. [105], proposed the use of shikonin (**11**) in combination with erlotinib (**23**), a well-known anticancer drug, which competes for the ATP-binding site of the tyrosine kinase (TK) domain in EGFR.

On a different note, Khaw et al. [86], focused their studies on plumbagin (**8**) and its telomerase activity in human glioblastoma multiforme cells [A172, KNS60, U251MG(KO)] and medulloblastoma cells (ONS76). Telomerase is a reverse transcriptase specialized in synthesizing telomeric DNA, thus, contributing to maintaining functional telomeres [106]. It has differential expression in normal and cancer cells, which makes it an attractive new target for chemotherapy. Validation of mechanisms already reported for plumbagin (**8**), such as cell cycle arrest at the G2/M phase and report of repression of telomerase activity and shortening of telomeres after incubation with the compound for 15 days, was carried out by this group.

**Fig. 10:**
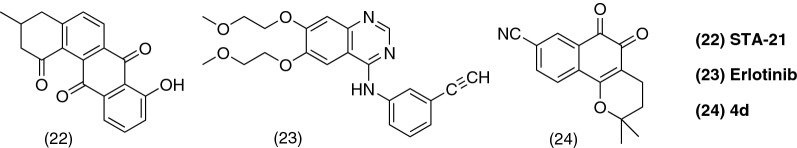
Chemical structures of STA-21, erlotinib and 4d, a compound which acts as MALT1 inhibitor. A summary of all the mechanisms of action gathered in this review for naphthoquinones as anticancer agents can be seen in Fig. 11

Lim et al. [107], embarked on the search of mucosa-associated lymphoid tissue lymphoma translocation protein (MALT1) inhibitors in order to find new drugs for the treatment of diffuse large B-cell lymphoma, one of the most aggressive types of cancer with unmet clinical needs. They found that 1,2-amino-naphthoquinones were good inhibitors but lacked the ability to inhibit the proliferation of OCI-LY3 cells (human B-cell lymphoma) in vitro. When testing β-lapachone (**12**), in the search for new scaffolds, they interestingly found that it is a rather potent inhibitor of MALT1 (IC_50_ = 1.9 µM) and, as mentioned before, is an important inhibitor of cell growth. They also suggested the formation of a covalent bond between the enzyme and the compound. When assessing derivatives of β-lapachone (**12**) synthesized by the group, the addition of an electron-withdrawing substituent at the C8 position led to an increase in activity. The initial hit for this discovery was obtained through high throughput screening and subsequently modified through structure-based drug design strategies. Compound 4d (**24)** (Fig. 10) exhibited good antiproliferative activity, comparable to that of its precursor, and inhibited the paracaspase activity of MALT1, showing that the 1,2-naphthoquinone moiety could be a good starting point for the development of new MALT1 inhibitors in the future.

### Examples of recently synthesized naphthoquinones with enhanced anticancer properties

**Fig. 11:**
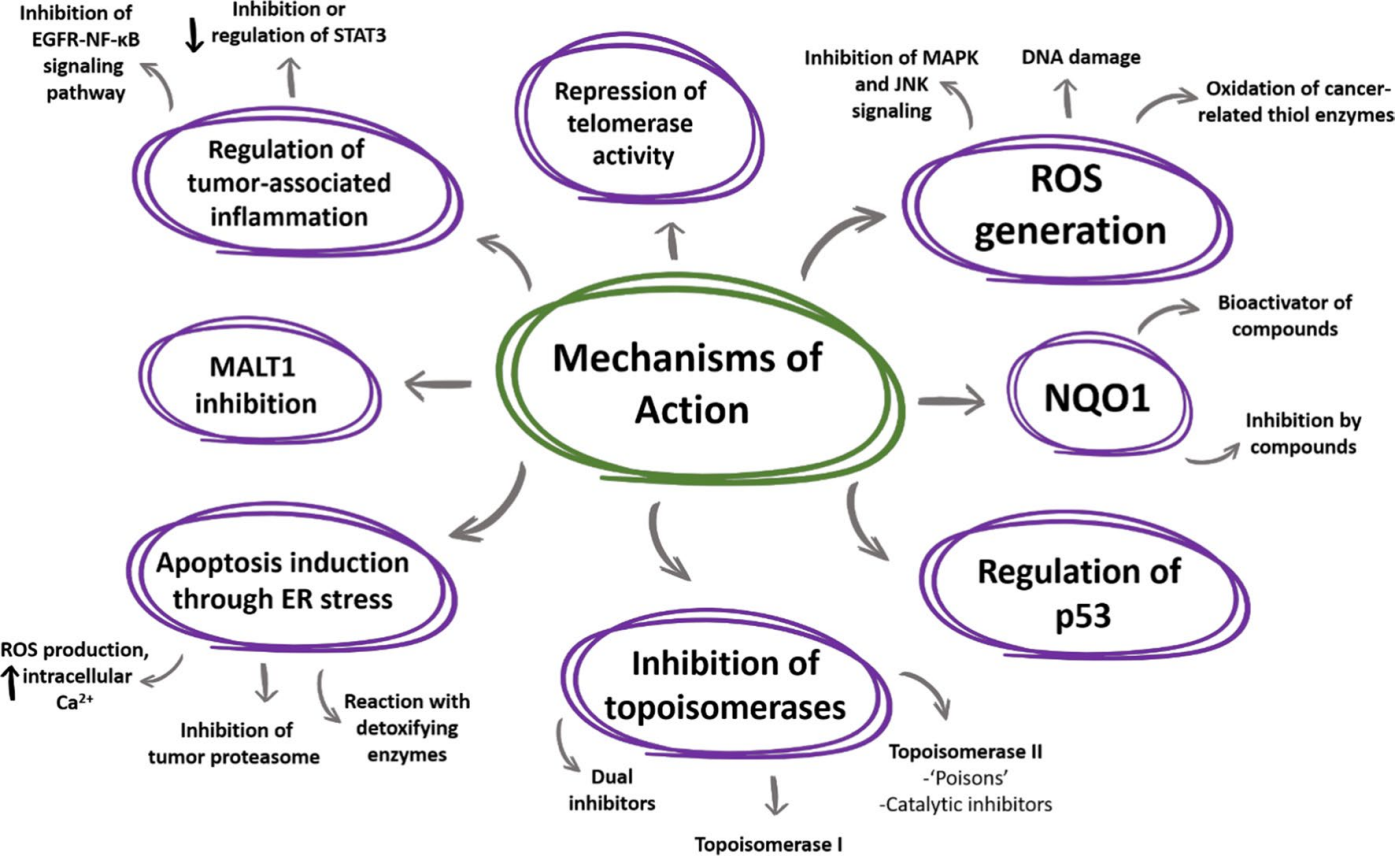
Summary of the mechanisms of action for naphthoquinones as anticancer agents gathered in this review

**Fig. 12:**
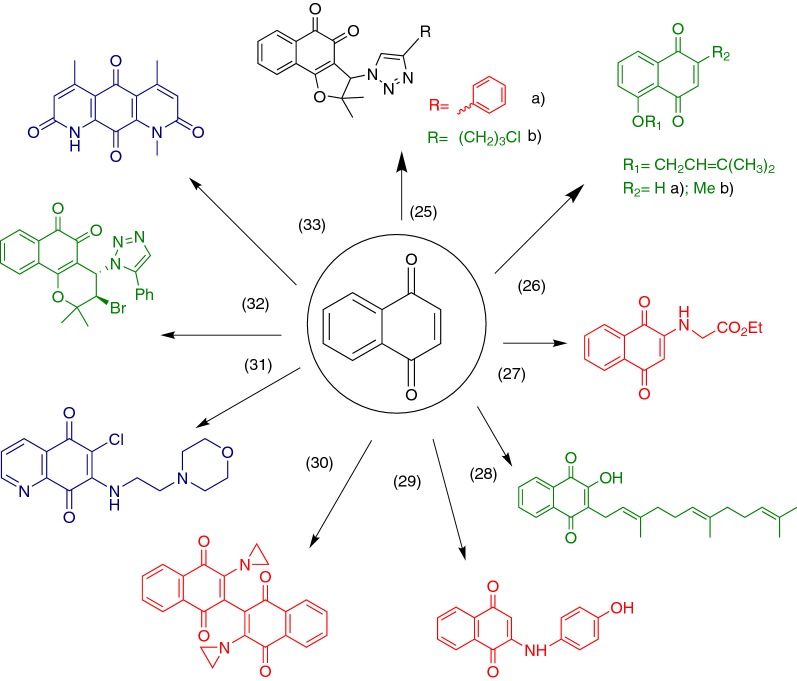
Novel structures found in the literature aimed to enhance the natural properties of naphthoquinones. Green—potency enhanced. Red—selectivity enhanced. Blue—enzyme inhibitors

As noted in this review, naphthoquinones as anticancer agents exhibit many mechanisms of action (Fig. 11) and it appears that there are more to be elucidated and/or clarified. Many groups of scientists around the world have embarked on the search for new analogous compounds which could enhance the action of the naturally-occurring naphthoquinones and provide new information into the actual mechanisms, or combinations of them, in which they exert their antitumoral action. Here, we chose some of the most notable and creative efforts found in the literature, which provide an advantage in potency or selectivity (sometimes in both), or work as ligands of specific targets described in previous sections. These molecules, summarized in Fig. 12, could be used as starting points for modifications which could turn them into better candidates and help in the further understanding of this pharmacological class.

Compounds **25a** and **25b** were synthesized by Cardoso et al. in 2014 [108] as part of a series of 1,2-furanonaphthoquinones linked to 1,2,3-1*H*-triazoles with the objective of investigating their antileukemic activity in lymphoid (MOLT-4 and CEM) and myeloid (K562 and KG1) leukemia cell lines The former has good potential for further studies since it turned out to be a potent and selective hit towards cancer cell lines also showing preference against leukemia lymphoid cell lines. Compound **25b** is also a promising hit with high potency as a cytotoxic agent (IC_50_ for leukemia cell lines range between 0.05 and 11.07 µM).

To develop new compounds based on the structures of juglone (**10**) and plumbagin (**8**), Fiorito and collaborators synthesized a series of derivatives from which compounds **26a** and **26b** resulted as the best candidates. **26a** is an ether derivative of juglone (**10**) which showed more cytotoxicity than its parent compound in 3 out of 6 cell lines tested (A549, SKMEL-28 melanoma and U373 glioblastoma). On the other hand, **26b** is also an ether derivative but of plumbagin (**8**), showing better potency than its precursor in all cell lines tested. To be noted, all the plumbagin (**8**) derivatives were more active than the ones related to juglone (**10**) suggesting that the presence of the methyl group on the position 2 of the naphthoquinones moiety is vital for cytotoxic activity [109].

A series of novel 2-N-aminonaphthoquinones were synthesized by de Moraes and collaborators, from which compound **27** was identified as the most relevant due to its yield in the synthesis (60%) and its high potency against a panel of different cancer cell lines (exhibiting 100% growth inhibition in MDAMB-435 breast carcinoma cells). It also showed good selectivity towards cancer cells when tested in normal peripheral blood mononuclear cells (PBMC), greater than some of the natural naphthoquinones used as precursors, such as juglone [110] (**10**).

Wang and collaborators synthesized a series of lipophilic lawsone (**7**) and juglone (**10**) derivatives, being compound **28** the most potent cytotoxic agent of the series showing the highest effect on the human colorectal adenocarcinoma cell line HT-29 with an IC_50_ of 2.0 µM in 48 h. They also reported that **28** arrested the cell cycle of these cells at the S phase and that it induced apoptosis [111].

Another novel series of aminonaphthoquinones was synthesized by Benites and collaborators in 2010, from which compound **29** was the best hit from cytotoxicity studies with human cancer cell lines of breast (MCF-7), prostate (DU145) and urinary bladder (T24), making it a promising candidate for further investigation [112]. Years later, in 2016, the same group reported that juglone (**10**) and **29** can be used in combination with ascorbate to potentiate their oxidative stress action in vivo in Ehrlich ascites tumor-bearing mice, showed inhibition of tumor progression [113].

In the search for new anti-HIV agents, Carter-Cooperet al. synthesized a novel series of amino dimeric naphthoquinones with reportedly good anti-integrase and cytotoxic activity against a panel of leukemia cancer cell lines [114]. Trying to increase their antitumoral properties, they made some modifications to the original structures that led to a series of bis-aziridinyl compounds. The derivative **30** showed the best results against acute myeloid leukemia (AML) cell lines (MOLM-14, MV4-11 and THP-1) with IC_50_ values ranging from 0.18 to 1.05 μM. This compound also had moderate selectivity towards normal cells from hematopoietic bone marrow, with selectivity index (SI) ranging from 1.5 to 2.

The high potency of *para*-quinone **31** caught the attention of Gopinath and collaborators when testing naphthoquinones for their action as inhibitors of USP2 through ROS production. Until this finding, *ortho*-quinones were regarded as good inhibitors of this cancer-related enzyme as opposed to *para*-quinones, precisely for their inability to behave as redox cyclers. This opens a door in this particular field of study for the development of new para-quinones which can target USP2 [59].

While looking for new trypanocidal agents derived from quinone moieties, Bahia and collaborators synthesized a series of 1,2,3-triazoles and evaluated their cytotoxic activities in a panel of cancer cell lines from different human tissues as well on normal cells (peripheral blood mononuclear cells). Compound **32** was one of the most potent in various cancer cell lines with IC_50_ values ranging from 0.41 to 1.59 μM. It also showed good selectivity with an SI of 0.8–3.0 in comparison to that presented by a well-known and widely-used anticancer drug, doxorubicin (**2**), which was 0.8 [115].

Finally, deoxynyboquinone (**33**) was originally synthesized while studying the antibiotic nybomycin [116] and it has been reported to have a tenfold superior efficacy compared to that of β-lapachone (**12**) in killing different cancer cell lines in a NQO1-dependent manner [117]. Kolossov and collaborators further studied this compound to conclude that it is a more specific substrate of NQO1 and offered proof that it does not elicit off-target responses at effective concentrations, unlike β-lapachone (**12**) [77].

**Fig. 13:**
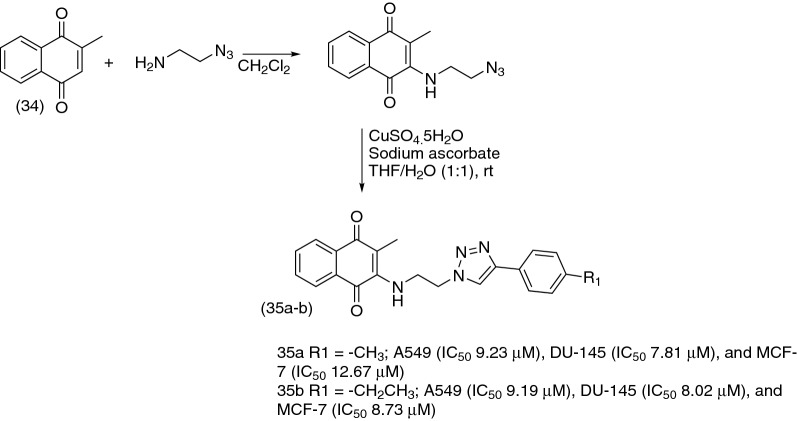
Synthesis of menadione-1,2,3-triazole hybrids

Another simple naphthoquinone that exhibits anticancer activity and is also widely used as starting material for the preparation of derivatives with cytotoxic potential is menadione (**34**). In 2018, Prasad and co-authors [118] synthesized a series of novel menadione-based triazole hybrids (Fig. 13) and evaluated their anticancer activity against five selected cancer cell lines including lung (A549), prostate (DU-145), cervical (Hela), breast (MCF-7), and mouse melanoma (B-16). They found that the majority of hybrid compounds displayed significant anticancer activity with special attention for compounds (**35a**) and (**35b**), which exhibited potent activity against all cell lines.

**Fig. 14:**
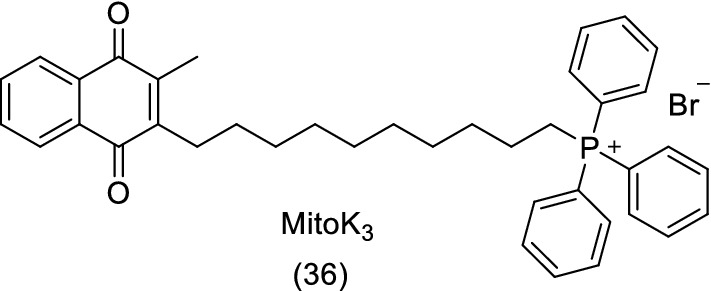
Chemical structure of MitoK_3_

Oliveira and collaborators [119], also used menadione to prepare a mitochondrial-directed agent, MitoK3 (**36**), which was developed by conjugating a TPP (triphenylphosphonium) cation to the C3 position of menadione’s naphthoquinone ring, increasing its selective accumulation in mitochondria as well as leading to alterations of its redox properties and consequent biological outcome (Fig. 14). MitoK3 showed cytotoxic activity towards human cancer cell lines of liver (HepG2), breast (MCF-7) and lung (A549). Moreover, it also enhanced the cytotoxic properties of doxorubicin (**2**) in A549 cells.

### Are naphthoquinones as PAINful as they seem?

Not all the attention which naphthoquinones have drawn to themselves has been on the bright side. Since the publishing of a highly cited article written by Baell and Holloway 7 years ago [120], which created many followers worldwide, naphthoquinones have been disregarded and looked upon by a great portion of the scientific community.

In the referred article, the authors coined the term pan-assay interference compounds (PAINs) to refer to compounds which would form aggregates, react with proteins or interfere in screening assays, leading to false positives [120]. In an attempt to prevent scientists from pursuing studies with them, they created a set of computational filters (alerts) which have been widely accepted by academia and the pharmaceutical industry, that allows for the recognition of certain substructures within molecules that are regarded as PAINs. A set of 480 PAINs alerts were created aiming at identifying and discarding these substructural features frequently found in them and suggesting that they could be used to flag false screening hits and annotate suspect compounds in screening libraries [120]. Particularly, in an article published in 2016, the main author maintained the idea, extending their application to natural products and referred to quinones as compounds which tend to be redox active as well as reactive to nucleophiles such as the amino acids cysteine and lysine, present in the side chains of proteins. They regarded their screening hits as PAINs and nonprogressable [121].

Although what was proposed by Baell and Holloway is to some extent, true, in the sense that when working with compounds, which have some of these characteristics, scientists should be careful enough not to report false/biased results in order to avoid irreproducibility in research, ruling them out of any kind of structural optimization campaign or from the pharmaceutical pipeline could lead to the loss of valuable chemical material. Still, the question remains of which molecules should be considered as PAINs [122].

In an effort to ameliorate the blind application of these filters, some authors have published data that question their usefulness. One finding which is crucial is the fact that many PAINs substructures can also be found in drugs available in the market [123, 124], meaning that if the filters are used without rational thinking, potentially good drug candidates can be disregarded. Phenotypic assays, which are regaining the value they used to have in the past for screening libraries of compounds since they take the biological setting as a whole, would be incredibly affected by the use of PAINs filters, preventing optimization of the structure, and again, leading to the loss of possible candidates [123].

Also, Capuzzi et al., have recently published a study in which they hypothesized that PAINs filters have limited extrapolative power. They based on the limitations of the original study, in which a relatively small library of compounds of a proprietary nature was used, meaning that complete chemical structures were not shown, and they were tested for just one type of activity (protein–protein interaction inhibition) in only six HTS campaigns, using a single detection technology, AlphaScreen [122]. They could prove that many compounds which contain PAINs alerts do not exhibit assay promiscuity and they urge the scientific community not to use these filters without conducting orthogonal experimental assays. They concluded, through the use of public data, that many compounds without PAINs alerts are actually more promiscuous than the ones identified by the filters [122]. These findings are supported by another recent study indicating that the molecular environment and structural context in which the putative PAIN substructure is can play a fundamental role whether its parent compound can act as an artifact or not [125].

Finally, something that cannot be overlooked is the potential application of this kind of compounds which elicit various pharmacological actions, in the advancing field of polypharmacology. Accordingly to this concept, compounds which have the ability to interact with multiple targets are actually desired, and this kind of versatility should be clearly distinguished from promiscuity that derives from non-selective activity [126].

## Conclusions

Naphthoquinones act as anticancer agents through a variety of mechanisms involved in all types of cancer. Some of the most important explored so far and which have been extendedly reported in the literature are: damage of DNA through generation of ROS, inhibition of topoisomerase II, regulation of the tumor suppressor factor p53 and induction of apoptosis via ERS. In this review, we summarized the most well-known mechanisms as well as those which have been recently described in the literature, providing new interesting targets to be researched in this pharmacological family. There is still a lot to be elucidated about the ways in which these compounds exert their action as anticancer agents and how to enhance their activity in order to exploit their potential to the maximum. Being privileged structures in pharmacology and chemistry, naphthoquinones will keep on fascinating scientists around the globe and will be further studied and researched in the many fields they have the potential to thrive.

## Data Availability

Not applicable.

## Abbreviations

ADMET: absorption, distribution, metabolism, excretion and toxicity
AIF: apoptosis-inducing factor
AML: acute myeloid leukemia
ATF6: activating transcription factor 6
Bax: apoptosis regulator BAX
Bcl-2: B cell lymphoma 2 protein
CAD: caspase-activated DNase
Cdks: cyclin-dependent kinases
EGFR: epidermal growth factor receptor
ER: endoplasmic reticulum
ERS: endoplasmic reticulum stress
FAD: flavine adenine dinucleotide
IAP: inhibitor of apoptosis proteins
IC50: 50% cytotoxic concentration
ICBP90: inverted CCAAT box binding protein of 90 kDa
iNOS: inducible nitric oxide synthase
IRE1α: inositol-requiring enzyme 1
JNK: c-jun-NH2-kinase
MALT1: mucosa-associated lymphoid tissue lymphoma translocation protein
MAM: 2-methoxy-6-acetyl-7-methyljuglone
MAPK: mitogen-activated protein kinase
MIX: mitoxantrone
MNQ: 2-methoxy-1,4-naphthoquinone
NADH: nicotinamide adenine dinucleotide reduced
NADPH: nicotinamide adenine dinucleotide phosphate reduced
NCDs: non-communicable diseases
NF-κB: nuclear factor kappa B
NO: nitric oxide
NQO1: NADPH-quinone oxidoreductase 1
PAINs: pan-assay interference compounds
PBMC: peripheral blood mononuclear cells
PERK: pancreatic endoplasmic reticulum kinase
PUMA: Bcl-2-binding component 3
ROS: reactive oxygen species
SEA: similarity ensemble approach
SI: selectivity index
STAT: signal transducer and activator of transcription
TK: tyrosine kinase
TNF: tumor necrosis factor
TP53: tumor protein p53
TPP: triphenylphosphonium
UGT: uridine diphosphate-glucuronosyltransferase
UPR: unfolded protein response
USP: ubiquitin-specific proteases
